# Primary Isolation Strain Determines Both Phage Type and Receptors Recognised by *Campylobacter jejuni* Bacteriophages

**DOI:** 10.1371/journal.pone.0116287

**Published:** 2015-01-13

**Authors:** Martine C. Holst Sørensen, Yilmaz Emre Gencay, Tina Birk, Signe Berg Baldvinsson, Claudia Jäckel, Jens A. Hammerl, Christina S. Vegge, Horst Neve, Lone Brøndsted

**Affiliations:** 1 Department of Veterinary Disease Biology, University of Copenhagen, Frederiksberg, Denmark; 2 National Food Institute, Technical University of Denmark, Søborg, Denmark; 3 Department Biological Safety, Federal Institute for Risk Assessment, Berlin, Germany; 4 Department of Microbiology and Biotechnology, Max-Rubner Institut, Federal Research Institute for Nutrition and Food, Kiel, Germany; Centro Nacional de Biotecnologia - CSIC, SPAIN

## Abstract

In this study we isolated novel bacteriophages, infecting the zoonotic bacterium *Campylobacter jejuni*. These phages may be used in phage therapy of *C. jejuni* colonized poultry to prevent spreading of the bacteria to meat products causing disease in humans. Many *C. jejuni* phages have been isolated using NCTC12662 as the indicator strain, which may have biased the selection of phages. A large group of *C. jejuni* phages rely on the highly diverse capsular polysaccharide (CPS) for infection and recent work identified the *O*-methyl phosphoramidate modification (MeO*P*N) of CPS as a phage receptor. We therefore chose seven *C. jejuni* strains each expressing different CPS structures as indicator strains in a large screening for phages in samples collected from free-range poultry farms. Forty-three phages were isolated using *C. jejuni* NCTC12658, NCTC12662 and RM1221 as host strains and 20 distinct phages were identified based on host range analysis and genome restriction profiles. Most phages were isolated using *C. jejuni* strains NCTC12662 and RM1221 and interestingly phage genome size (140 kb vs. 190 kb), host range and morphological appearance correlated with the isolation strain. Thus, according to *C. jejuni* phage grouping, NCTC12662 and NCTC12658 selected for CP81-type phages, while RM1221 selected for CP220-type phages. Furthermore, using acapsular ∆*kpsM* mutants we demonstrated that phages isolated on NCTC12658 and NCTC12662 were dependent on the capsule for infection. In contrast, CP220-type phages isolated on RM1221 were unable to infect non-motile ∆*motA* mutants, hence requiring motility for successful infection. Hence, the primary phage isolation strain determines both phage type (CP81 or CP220) as well as receptors (CPS or flagella) recognised by the isolated phages.

## Introduction

Campylobacteriosis is the most commonly reported zoonosis and the leading cause of human bacterial gastroenteritis in EU [1]. Contaminated poultry meat is the major source of *Campylobacter jejuni* infections and in some countries up to 90% of these domestic birds are colonized and carry this human pathogen as a part of their normal intestinal flora [1]. A number of studies report the application of bacteriophages (phage therapy) to reduce the number of *C. jejuni* either at the poultry farm setting [2–6] or post-slaughter on the chicken meat [7–9] with significant levels of reduction. Thus, the use of phages is a promising intervention strategy. However, phage treatment against *C. jejuni* can only be efficient if the phages applied are capable of infecting the diverse population of *C. jejuni* strains found in the poultry gut or on the contaminated meat [10].

Attachment of a phage to a specific receptor on the surface of the bacterial host is the first step in phage infection. Several studies have shown that either changes in the capsular polysaccharides (CPS) or loss of motility is involved in phage resistance development in *C. jejuni*, indicating that CPS and flagella may be the main receptors of phages infecting this bacterium [11–14]. Indeed, mutant analyses and adsorption assays showed that *C. jejuni* phage F336 binds to CPS, while motility is essential for successful infection by phage F341 [14,15]. Even though the receptor for phage F341 has not yet been identified, transmission electron images demonstrated that phage F341 indeed attaches to the flagella of *C. jejuni*. Our current hypothesis is that either the widespread flagellar *O*-linked pseudaminic acid or even the FlaAB proteins may be the receptor of this phage, since mutants lacking legionaminic acid and derivatives of pseudaminic acid attached to the flagella are susceptible to F341 infection [15].

CPS is the major determinants of the Penner serotyping scheme of *C. jejuni*, and currently more than 60 different serotypes are known, demonstrating the diversity of these surface components [16,17]. The CPS of *C. jejuni* are highly variable and complex, due to the many different carbohydrate backbones that also carry highly diverse modifications such as *O*-methyl, ethanolamine, aminoglycerol and *O*-methyl phosphoramidate (MeO*P*N) [18–24]. In addition, phase variable expression of these CPS modifications results in further variations, even among strains encoding the same genes in CPS loci [22,25,26]. The MeO*P*N modification is common within the *Campylobacter* genus and has been identified in ∼70% of *C. jejuni* isolates despite that phosphoramidates are rarely found in nature [27,28]. This commonality suggest an important biological role for this surface structure and loss of MeO*P*N on the *C. jejuni* cell surface is associated with a decrease in serum resistance, enhanced invasion of CaCO-2 cells and reduced colonisation in a piglet model [28]. Interestingly, we also identified the MeO*P*N moiety attached to Gal*f*NAc of CPS in *C. jejuni* NCTC11168 as a novel phage receptor of several lytic bacteriophages [13,14]. Development of phage resistance caused by changes in the CPS modifications is expected to be rather frequent, due to the phase variable nature of these components. This was confirmed by *in vivo* chicken colonization experiments where phage resistance by loss of the MeO*P*N receptor was easily achieved without causing a decline in colonization levels of *C. jejuni* [13]. Knockout MeO*P*N transferase mutants in *C. jejuni* strain 81–176 were also not affected in colonisation levels of chickens, further supporting a minor role of this surface modification in chicken colonisation [28]. Consequently, phage resistant development of *C. jejuni* may potentially affect the outcome of a phage treatment.

To develop an efficient phage therapy and prevent phage resistance development, it has been suggested to use phage cocktails that target multiple receptors as well as broadening the lytic spectrum of the applied phages [3,6,29]. Noteworthy, a large number of the previously identified and characterized bacteriophages are isolated using *C. jejuni* NCTC12662 as indicator strain due to its high sensitivity towards phages [4,30–38]. Indeed, almost all *Campylobacter* phages isolated so far are members of the *Myoviridae* family and can be categorised into three groups (group I, II and III) based on genome size and morphology [39]. The majority of these phages belong to the *Eucampyvirinae* subfamily, represented by group II and III phages, that is divided into two genera, the Cp220likevirus (180–190 kb genome, CP220-type phages) and the Cp8unalikevirus (130–140 kb genome, CP81-type phages) [40]. This extensive use of one particular indicator strain may have aided in the recovery of *Eucampyvirinae* phages that could be biased in respect to recognition of *C. jejuni* receptors. The current phage collections may thus not represent the phage diversity needed to target numerous receptors in the diverse population of *C. jejuni* expressing very different surface structures.

Here we isolated novel phages from free-range chicken faecal samples collected throughout Denmark using well characterized Penner serotyped indicator strains expressing different capsular structures and modifications. Our analysis showed that the isolation strain both influence the phage type isolated and the receptors recognised by these *C. jejuni* phages.

## Materials and Methods

### Bacterial strains and preparation of bacterial lawns

Strains used in this study are shown in Table 1. Preparations of bacterial lawns were done according to Sørensen et al, 2011 [14]. Briefly, standard growth condition of *C. jejuni* strains was performed by cultivation on blood agar Base II (Oxoid) supplemented with 5% calf blood (BA) followed by incubation under microaerobic conditions (6% CO_2_, 6% O_2_ and 88% N_2_H_2_) for 18–24 h at 37°C. For preparation of bacterial lawns, cultivated cells were harvested with cation adjusted (1 mM CaCl_2_ and 10 mM MgSO_4_) Brain Hearth Infusion broth (Oxoid) (CBHI). Bacterial suspensions were then adjusted to an optical density at 600 nm (OD_600_) of 0.35 (∼10^9^ cfu/ml) in CBHI and incubated microaerobically at 37°C for 4 h. Then, 500 μl of cultures were added to 5 ml of molten NZCYM overlay agar (NZCYM broth [Sigma] with 0.6% agar [Sigma]) tempered to 45°C and poured on previously prepared and dried NZCYM basal agar (with 1.2% agar [Sigma] and 10 μg/ml vancomycin [Sigma]) and allowed to dry for 45–60 min in a flow hood.

**Table 1:** Wild type and mutant strains used in this study.

***C. jejuni* strain**	**Origin**	**Penner serotype**	**CPS data from**	**Carbohydrates in the CPS backbone**	**CPS modifications**					**CPS references**
					**Me**	**O-Me**	**MeO*P*N**	**β-Xlu**	**O-Ac**	
NCTC12658	Not known [35]	HS1.44	ATCC43429	Galactose, glycerol, fructofuranose	−	−	+	−	−	[22]
104–733	Chicken [35]	HS1.44	ATCC43429	Galactose, glycerol, fructofuranose	−	−	+	−	−	[22]
NCTC11168	Human NCTC	HS2	NCTC11168	Ribofuranose, N-acetyl galactosamine, glucoruronic acid, aminoglycerol, Heptose	+	+	+	−	−	[24,27]
1447	Chicken [35]	HS4complex	CG8486	N-acetyl glucopyranosamine, heptopyranose	−	−	+	−	−	[18]
NCTC12662	Chicken [35]	HS5j	NCTC12662	ND	ND	ND	+	ND	ND	[43]
81116	Human NCTC	HS6	81116	Glucose, glucuronic acid, mannose, galactose, N-acetylglucosamine	−	−	−	−	+	[23]
81–176	Human NCTC	HS23.36	81–176	N-acetylglucosamine, galactose, *altro*-heptose	+	−	+	−	−	[21,59]
RM1221	Chicken [62]	HS53	RM1221	6d-*manno*-Heptose	−	−	−	+	−	[19]
NCTC12568*∆kpsM*	[15]	ND	ND	ND	ND	ND	ND	ND	ND	
NCTC12658*∆motA*	[15]	ND	ND	ND	ND	ND	ND	ND	ND	
NCTC12662*∆kpsM*	This study	ND	ND	ND	ND	ND	ND	ND	ND	
NCTC12658*∆motA*	This study	ND	ND	ND	ND	ND	ND	ND	ND	

ND: Not determined, Me: Methyl, O-Me: *O*-methyl, MeO*P*N: *O*-methyl phosphoramidate, β-Xlu: Xylose, O-Ac: O-acetyl.

NCTC: National Collection of Type Cultures.

### Isolation, purification and propagation of phages

**Figure 1:**
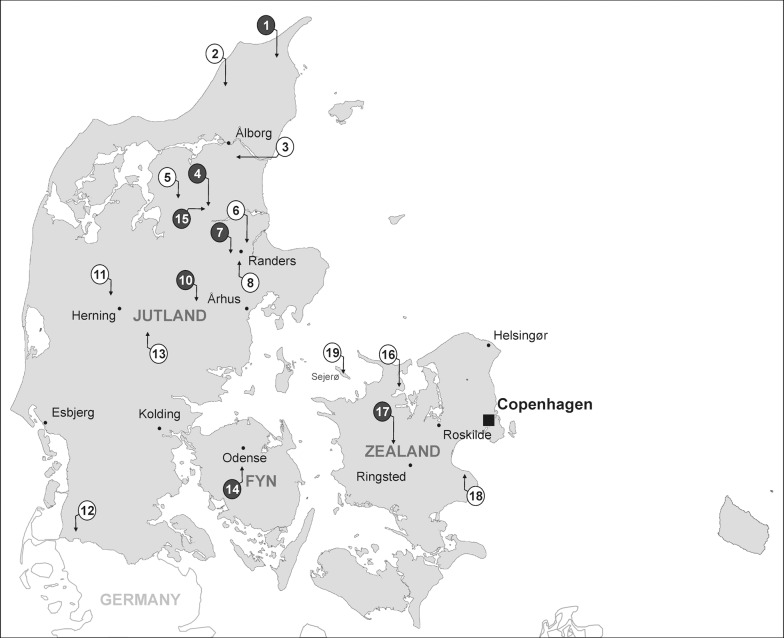
Distribution of sampled farms indicated by number in circles located throughout Denmark. Black circles represent farms where phages could be isolated.

A total of seventeen free-range chicken farms spread throughout Denmark (13 in Jutland; 1 in Fyn; 3 in Zealand) and one goose farm (Sejerø) were visited in September 2011 (Fig. 1) and fresh faecal samples were collected from five parts of each farm in a plastic bag and taken into analysis in less than 96 hours. As there was no direct contact with the animals and fecal samples were collected randomly from the housing environment without disturbing the daily routines of the birds, formal approval of this study as an animal experiment was not necessary according to Danish law. Sampling was carried out on private land and permission was granted by the farm owners in return of being anonymous. In the laboratory up to five-gram aliquots of the fecal samples were aseptically collected in a 50 ml tube and a 1:10 dilution with sterile SM buffer (100 mM NaCl [Merck], 8 mM MgSO_4_.7H_2_O [Merck], 0.01% gelatine [Sigma], 50 mM Tris-HCl [Sigma] in 1 l of distilled water with pH 7.5) was made and left overnight at 4°C on a gyratory shaker (100 rpm) for dissociation of phages. After thorough vortexing, the resulting upper phases were taken in sterile tubes and centrifuged at 3.000 *g* for 5 min for removal of debris. Supernatants were syringe filtered (0.2 μm filters) and droplets of five times 10 μl were applied on prepared lawns of all indicator strains. Following the settling of droplets, plates were incubated microaerobically at 37°C for 24 h and analysed for the presence of plaques. If plaques were observed the plaque morphologies were recorded.

For purification of phages, single plaques with varying morphologies were transferred to 100–500 μl of SM buffer with sterile pipette tips, vortexed thoroughly and tenfold serial dilutions were prepared and applied on a lawn of the original indicator strain. Following at least three cascades of purification for each plaque, phages were propagated for preparation of phage stocks. Phage propagation was performed on the lawns of the isolating strains by the plate lysis method as described elsewhere [13] and stocks were stored at 4°C.

### Titration, host range and number of plaques

Titration and host range determination of phages by spot assay were conducted as described previously [13]. Briefly, *C. jejuni* strains were cultivated under standard growth conditions, harvested into CBHI and adjusted to OD_600_ of 0.35. The bacterial suspensions were then incubated for 4 h at 37°C under microaerobic conditions and bacterial lawns were made as already described. Tenfold serial dilutions of the phage stock suspensions in SM buffer up to 10^−7^ were made and three times 10 μl of each dilution and the undiluted stock suspension were spotted on the bacterial lawns of the investigated strains. Following incubation under microaerobic conditions for 18–24 h at 37°C, plaque morphologies were observed, plaques counted and mean plaque forming units per ml (pfu/ml) was calculated for each strain. The data represented are the means from between 2–8 independent experiments.

### Pulse Field Gel Electrophoresis (PFGE) analysis

PFGE analysis for characterization of phage genomes was done as described by Hansen et al. (2007) [35] with slight modifications. Briefly, phage stock suspensions (10^7^–10^9^ pfu/ml) were mixed either 1:1, 2:1 or 8:1 with 1%, 2.0% or 4% SeaKem Gold agarose respectively, prepared in TE buffer (10 mM Tris and 1 mM EDTA [pH 7.2]) and liberated overnight in lysis buffer (10 mM Tris, 100 mM EDTA, 1% sarkosyl and 0.1 mg/ml proteinase K) at 55°C with gentle shaking (100 rpm). Following digestion of phage capsids, blocks were washed 4–6 consecutive times (55°C, 100 rpm) for 20 min in washing buffer (20 mM Tris and 50 mM EDTA) and stored in TE buffer at 4°C until the PFGE was performed. Digestion of phage genomes with restriction endonuclease HhaI (New England BioLabs) was carried out, following preparation of 3–5 mm block slices, according to the Manufacturer and completed in 17–19 h at 37°C. For PFGE; intact or digested phage DNA harboring slices were loaded in 1% SeaKem Gold agarose along with low range PFGE Marker (New England BioLabs), run for 14 h at 6 V/cm, included angle 120, and switch time 2–10 s using Biorad CHEF-III apparatus. Finally, gels were stained for 30 min with ethidium bromide and washed in distilled water for 1 hour before being visualized manually.

### SmiI restriction profiling of phage genomes

Restriction profiles of the phage genomes were conducted as described by Hammerl et al. (2011) [41]. Phage DNA isolated by proteinase K/SDS treatment of CsCl-purified phages was used for restriction profiling. Restriction endonuclease SmiI was used according to Manufacturer’s recommendations (Fermentas) and analysed by agarose gel electrophoresis using standard procedures.

### Construction of *∆kpsM* and *∆motA* mutants

For the construction of the acapsular NCTC12662*∆kpsM* mutant strain, the pTA-3 vector (pTZ57R/T::*kpsM*::*aphA*, Kan^R^) containing an amplified *kpsM::kan* fragment [15] was introduced into the *C. jejuni* NCTC12662 by electroporation and transformants were selected for kanamycin resistance. The pCV1206-1 vector construct (pCR2.1-TOPO::∆*motA*::*cat*, Amp^R^, Kan^R^, Cam^R^) [15] used to generate the non-motile NCTC12662*∆motA* strain, was introduced into *C. jejuni* NCTC12662 by electroporation and transformants were selected for chloramphenicol resistance. All mutant constructs were verified by PCR amplification and sequencing. Additionally the *motA* deletion was also confirmed by complete loss of motility in a soft agar motility assay as described below. Attempts to construct *∆kpsM* RM1221 and *∆motA* RM1221 mutants were unfortunately unsuccessful.

### Motility assay

The motility assay was essentially performed as described previously [14] with minor modifications. Briefly, *C. jejuni* strains were grown under standard growth conditions on BA, harvested in BHI and OD_600_ was adjusted to 0.1. One μl of the adjusted cell suspension was placed in the centre of ten 0.25% heart infusion broth (Difco) agar plates pre-dried for 45 min. Plates were incubated under standard growth conditions and growth zones demonstrating the ability to move in the soft agar were measured after 24 and 48 hours. Measurements are given as the mean count standard deviations of two independent experiments.

### Transmission Electron Microscopy (TEM) of phages

The basic protocol of Ackermann (2009) [42] was modified for TEM analysis as follows. Ultra-thin carbon films prepared in a high vacuum coating system (BAL-TEC MED 020; Balzers, Lichtenstein) on a mica sheet were cut (~3×3 mm in size) and floated into a 100 μl drop of phage suspension. After 5–10 min adsorption, films were transferred into a drop of 1% (v/v) of EM-grade glutaraldehyde (20 min) and subsequently into a drop of 2% (w/v) uranyl acetate for negative staining (1–2 min). After two washes for a few seconds in drops of distilled water, samples were picked up with 400-mesh copper grids (Plano, Wetzlar, D). Electron micrographs were taken in a Tecnai 10 transmission electron microscope (FEI Company, Eindhoven, The Netherlands) at an accelerating voltage of 80 kV. Digital micrographs were taken with a Megaview G2 CCD camera (Olympus SIS, Münster, Germany).

## Results

### Phage isolation from free-range poultry farms

For the isolation of *C. jejuni* phages from free-range poultry farms, we used seven *C. jejuni* indicator strains belonging to different Penner serotypes, thus expressing different CPS carbohydrates and modifications of the CPS (Table 1). CPS has structurally been determined for Penner serotypes HS1.44, HS2, HS4, HS6, HS23.36, and HS53 represented by strains NCTC12658, NCTC11168, 1447, 81116, 81–176, and RM1221, respectively. In addition, the Penner serotype HS5j strain NCTC12662 was included, due to its high susceptibility to phage infection. The CPS carbohydrate backbone is not resolved for serotype 5j, but we determined, by high-resolution magic angle spinning NMR spectroscopy, that the CPS carries one or more MeO*P*N modifications [43]. This has recently been confirmed by the genome sequence of this strain identifying two MeO*P*N transferase homologues [44].

In order to isolate as many different *C. jejuni* phages as possible, all faecal samples were collected from free-range chicken and goose farms where the birds had constant interaction with the environment (Fig. 1). A total of 43 phages showing a variety of plaque morphologies were isolated, purified and propagated from seven of the eighteen free-range poultry farms (Fig. 1, Table 2). Interestingly, phages were only isolated using *C. jejuni* NCTC12662, NCTC12658 or RM1221 as the indicator strains, while no phages were isolated using the remaining strains (NCTC11168, 1447, 81116, and 81–176). The samples from farm 15 allowed isolation of phages using all three indicator strains, whereas the samples from farm 7 resulted in isolation of phages using strain NCTC12658 and NCTC12662. In contrast, from farms 1, 4, 10, 14, and 17, phages were only isolated using one of these three indicator strains (Table 2). Consequently, phage isolation was not achieved at all farms, and not all *C. jejuni* strains proved useful for phage isolation.

**Table 2:** Number of isolated and purified phages from free-range chicken farms.

		**Farm number**						
**Indicator strain**	**Penner serotype**	**1**	**4**	**7**	**10**	**14**	**15**	**17**
NCTC12658	HS1.44	2	-	2	-	-	2	-
NCTC11168	HS2	-	-	-	-	-	-	-
1447	HS4c	-	-	-	-	-	-	-
NCTC12662	HS5j	-	5	11	5	-	2	-
81116	HS6	-	-	-	-	-	-	-
81–176	HS23.36	-	-	-	-	-	-	-
RM1221	HS53	-	-	-	-	2	5	7

### Host range and genome size of isolated phages

Among the 43 purified phages, we identified 20 distinct phages based on the host ranges and genome restriction analyses (Table 3). Thus, phages originating from the same farm or flock and showing the same host range with similar plaque counts as well as having identical genomic restriction patterns were classified as being replicates of the same phage isolated more than once (Table 3). Although all phages initially were isolated using *C. jejuni* NCTC12662, NCTC12658 or RM1221 as indicator strains, most phages did infect additional strains such as NCTC11168, 81116, and 1447 that were also used in the isolation procedure (Table 3). Regardless of the initial isolation strain, all phages were able to infect *C. jejuni* NCTC12662 (Table 3) with high efficiency of plaquing (EOP), confirming the high sensitivity of NCTC12662 to phage predation. In contrast, none of the phages formed plaques on *C. jejuni* 81–176.

**Table 3:** Host range, genome size distribution and restriction profile of phages isolated from free-range poultry farms.

**Phage**	**Farm**	**Indicator strain used for isolation**	**Number of plaques (log_10_) formed on strain**								**Genome size determined by PFGE (∼kb)**	**Restriction profile**	**Distinct phages as indicated by number^c^**
			**NCTC 12658**	**104–733**	**NCTC 11168**	**1447**	**NCTC 12662**	**81116**	**81–176**	**RM1221**			
F347	1	NCTC12658	7.1	7.3	6.6	R	7.4	R	R	R	140	IIIa	1
F348	1	NCTC12658	8.0	8.0	7.3	R	8.4	R	R	R	140	IIIa	1
F349	7	NCTC12658	7.4	7.6	R	2.5	7.7	R	R	R	140	IIIb	2
F350	7	NCTC12658	7.8	7.3	R	2.3	8.2	R	R	R	140	IIIb	2
F351	15	NCTC12658	7.1	7.3	6.5	R	7.5	R	R	R	140	IIIg	3
F352	15	NCTC12658	7.8	7.7	6.9	1.5	8.0	R	R	R	140	IIIg	4
F353	4 (flock 1)	NCTC12662	7.5	7.8	R	R	8.6	R	R	R	140	IIId	5
F354	4 (flock 1)	NCTC12662	6.1	6.4	R	R	7.4	R	R	R	140	IIId	5
F355	4 (flock 1)	NCTC12662	5.7	7.0	R	R	7.9	R	R	R	140	IIId	5
F356	4 (flock 2)	NCTC12662	5.7	5.9	R	R	6.9	R	R	R	140	IIIe	6
F357	4 (flock 2)	NCTC12662	5.6	5.0	R	R	7.3	R	R	R	140	IIId	7
F358	7	NCTC12662	7.9	8.2	R	R	9.6	R	R	R	140	IIIb	8
F359	7	NCTC12662	7.9	7.9	5.8	R	9.0	R	R	R	140	IIIb	9
F360	7	NCTC12662	8.1	8.5	5.4	R	8.8	R	R	R	140	IIIb	9
F361	7	NCTC12662	8.3	8.7	7.6	R	9.3	R	R	R	140	IIIc	10
F362	7	NCTC12662	8.1	8.3	7.2	R	9.0	R	R	R	140	IIIc	10
F363	7	NCTC12662	6.6	6.8	4.2	2.3	7.2	R	R	R	140	IIIb	11
F364	7	NCTC12662	7.9	7.6	5.6	3.4	8.5	R	R	R	140	IIIb	11
F365	7	NCTC12662	7.5	7.3	5.7	3.0	8.3	R	R	R	140	IIIb	11
F366	7	NCTC12662	8.1	8.3	R	3.9	8.9	R	R	R	140	IIIb	2
F367	7	NCTC12662	8.7	8.9	6.8	4.3	9.4	R	R	R	140	IIIb	11
F368	7	NCTC12662	7.6	8.0	R	3.7	8.4	R	R	R	140	IIIb	2
F369	10 (flock 1)	NCTC12662	8.3^a^	R	R	R	7.2	R	R	R	140	IIIf	12
F370	10 (flock 1)	NCTC12662	4.1^b^	R	R	R	7.5	R	R	R	140	IIIf	12
F371	10 (flock 1)	NCTC12662	R	4.1	R	R	8.5	R	R	R	140	IIIf	13
F372	10 (flock 2)	NCTC12662	7.7^b^	R	R	R	8.2	R	R	R	140	IIIf	14
F373	10 (flock 2)	NCTC12662	5.0^b^	R	R	R	8.4	R	R	R	140	IIIf	14
F374	15	NCTC12662	6.0	6.4	5.5	R	7.0	R	R	R	140	IIIg	3
F375	15	NCTC12662	9.1	9.3	9.6	3.4	9.4	R	R	R	140	IIIg	4
F376	14	RM1221	R	7.2	R	R	8.0	R	R	8.5	190	IIa	15
F377	14	RM1221	R	7.9	R	R	9.4	R	R	8.3	190	IIa	15
F378	15	RM1221	R	8.7	R	R	8.4	7.6	R	8.3	190	IIb	16
F379	15	RM1221	R	8.5	R	R	9.8	7.0	R	9.1	190	IIb	16
F380	15	RM1221	R	R	R	7.2	7.2	7.3	R	7.4	190	IIc	17
F381	15	RM1221	R	R	R	R	7.0	6.8	R	7.5	190	IId	18
F382	15	RM1221	R	R	R	R	7.2	6.8	R	7.1	190	IId	18
F383	17	RM1221	R	R	R	5.0	7.3	6.9	R	7.2	190	IIe	19
F384	17	RM1221	R	R	R	6.5	7.8	6.8	R	7.6	190	IIe	19
F385	17	RM1221	R	R	R	5.0	8.0	7.1	R	8.1	190	IIe	19
F386	17	RM1221	R	R	R	7.1	8.9	8.3	R	8.7	190	ND	19
F387	17	RM1221	R	R	R	5.0	8.8	7.6	R	8.4	190	ND	19
F388	17	RM1221	R	R	R	7.2	9.6	8.1	R	9.3	190	IIe	20
F389	17	RM1221	R	R	R	7.3	8.8	7.9	R	8.7	190	ND	19

R: resistant (determined as no plaque formation); ND: Not determined.

^a^Very small plaques were only observed in 1 out of 3 experiments.

^b^Very small plaques only observed in 1 out of 4 experiments.

^c^20 distinct phages were found based on host range and restriction profile. Phages isolated from the same farm or flock that showed the same host range and restriction profile are predicted to be replicates of the same phage isolated more than once and are given an identical number between 1 and 20.

**Figure 2:**
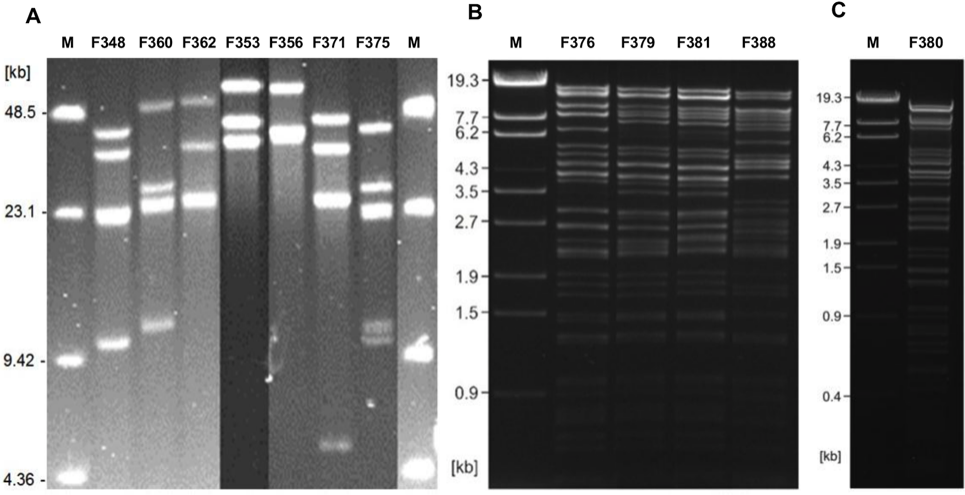
Restriction patterns of digested phage genomes representing different profiles. A. HhaI profiles of group III (∼140 kb) phage genomes. Lane 1: Marker (New England Biolabs), lane 2: Phage F348 (Type IIIa), lane 3: Phage F360 (Type IIIb), lane 4: F362 (Type IIIc), lane 5: Phage F353 (Type IIId), lane 6: Phage F356 (Type IIIe), lane 7: Phage F371 (Type IIIf), lane 8: Phage F375 (Type IIIg), and lane 9: Marker (New England Biolabs). B. SmiI profiles of group II (∼190 kb) phage genomes. Lane 1: Marker (DNA marker λ Eco130I), lane 2: Phage F376 (Type IIa), lane 3: Phage F379 (Type IIb), lane 4: Phage F381 (Type IId), lane 5: Phage F388 (Type IIe). C. SmiI profile of phage F380 (Type IIc): Lane 1: Marker (DNA marker λ Eco130I), lane 2: phage F380.

Interestingly, phage genome sizes corresponded to the indicator strain used as phages isolated on *C. jejuni* NCTC12658 and *C. jejuni* NCTC12662 all harboured genomes of approximately 140 kb, whilst phages isolated using *C. jejuni* RM1221 all were close to 190 kb in size (Table 3). Thus, all the isolated phages fall within the two *C. jejuni* phage groups, group III (130–140 kb genome, CP81-type phages) and group II (180–190 kb genome, CP220-type phages) described previously [39,40]. Restriction profiles of phage genomes revealed that they were highly different and could be categorised into a number of distinct groups that fell within the farm origin (Table 3, Fig. 2). Noteworthy, phages isolated from flock 1 on farm 4 showed one distinct restriction profile, whereas flock 2 from the same farm promoted the isolation of an additional phage with a different profile. Phages isolated from two different flocks on farm 10 showed the same restriction profile regardless of flock origin. Thus, even though the phages were isolated from the same farm they could be very different, which was particularly apparent for farm 15 (Table 3).

### Phage morphology

**Figure 3:**
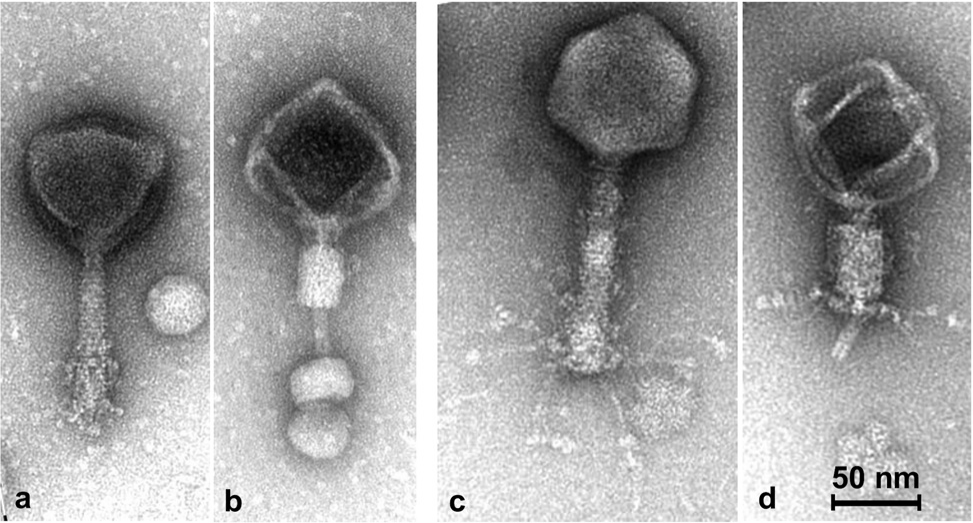
Electron micrographs of *C. jejuni* bacteriophages F362 (a and b) and F386 (c and d). a. Normal phage particle with extended tail and folded-up fibers. b. Phage particle with contracted tail sticking in small globular vesicles. Note the tail fibers are no longer visible. c. Normal phage particle with extended tail and flexible fibers with attached small terminal globular structures. d. Phage particle with contracted tail. Note tail fibers are still visible. Phages were stained with 2% uranyl acetate and examined on a Tecnai 10 transmission electron microscope.

Five phages (F348, F362, F375, F376 and F386) isolated from different farms and on different *C. jejuni* strains were chosen for analysis by transmission electron microscopy as representative of the 20 distinct phages. This analysis showed that all five phages belong to the *Myoviridae* family having icosahedral heads and contractile tails with morphology resembling other isolated *C. jejuni* phages [4,30–32,35–38,40,45]. Electron micrographs of a representative group III phage (F362) are shown in Fig. 3 (a and b) illustrating the phage morphology (head diameter: 99 ± 4 nm; n = 22, extended tail length: 107 ± 5 nm; n = 10). Phage particles with contracted tails and empty capsids were frequently sticking in small globular vesicles (most likely bacterial remnants) with their central thin tail tube. An undefined number of flexible tail fibers were detected to be intimately attached to the distal part of the extended tails covering approximately 1/3 of the total tail length in the “folded-up” position. However, in phage particles with contracted tails, these fiber structures are no longer visible. In Fig. 3 (c and d) representative micrographs are shown for the group II phages (F386). Although also belonging to the *Myoviridae* family, the morphological details differed remarkably from those of the group III phages with respect to the tail size (extended tail length: 140 ± 3 nm; n = 15) and orientation of the tail fibers. The flexible fibers of group II phage F386 were approximately 100 nm in length with small terminal globular structures and were generally expanded uniformly around the tail tip, while tail fibers of phage F362 were sticking on the distal tail regions (Fig. 3). After tail contraction, the tail fibers of F386 were still visible at the distal region of the contracted tail sheaths. Notably phage F386 (genome size 190 kb) has the same capsid dimensions (head diameter: 99 ± 5 nm; n = 15) as phage F362 (genome size: 140 nm) possibly indicating an elevated pressure within the capsid of the group II phages. This correlates with our observations of a significantly higher structural instability of group II phage capsids, as the number of intact F376 and F386 phages were low during microscopy. Thus, these phages fall into the category of the two phage types, the CP81-type (group III phages) and the CP220-type (group II phages), recently described for *C. jejuni* phages who belong to the proposed genera “CP8unalikevirus” and “Cp220likevirus” within the subfamily *Eucampyviriniae* [40].

### The role of CPS for phage infection

Looking closer into the host range of the two major groups of phages, we found that the isolation strain used also had a profound effect on the host range of the isolated phages. Many of the phages isolated using *C. jejuni* NCTC12662 and NCTC12658 could also infect *C. jejuni* 104–733, while a few phages infect *C. jejuni* NCTC11168 and 1447. However, none of these phages infect strain RM1221 and 81116 (Table 3). The sugar backbones of the CPS of all strains are very different, but NCTC12662, NCTC12658, 104–733, NCTC11168 and 1447 all carry the MeO*P*N modification of the CPS (Table 1), which has previously been identified as a receptor of a large group of capsular phages [13,14]

In contrast, phages isolated using *C. jejuni* RM1221 as indicator strain clearly displayed a different host range than the phages isolated using *C. jejuni* NCTC12662 and NCTC12658 (Table 3). The most prominent difference is that most of these phages also infect *C. jejuni* 81116 having a very different CPS structure compared to RM1221 (Table 1 and 3). Comparing the backbone sugars and the modifications of the CPS it is not possible to find any similarities between *C. jejuni* RM1221 and 81116 (Table 1), suggesting that these phages may be independent of CPS for infection.

**Figure 4:**
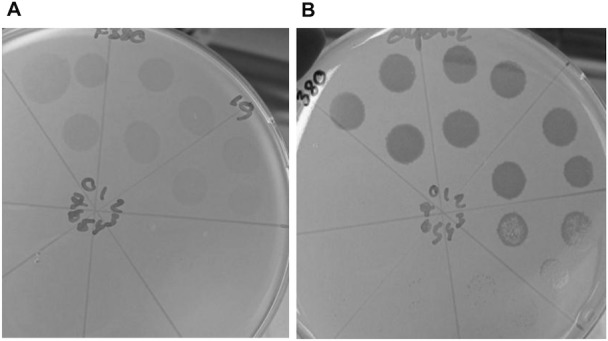
Plaque assay of phage F380 on strain A. NCTC12662 and B. NCTC12662*∆kpsM*. Clearer lysis and plaque formation can be seen on the acapsular version of the *C. jejuni* strain.

To investigate this further, we constructed an acapsular mutant of *C. jejuni* NCTC12662 by inserting a kanamycin resistance cassette into the *kpsM* gene and subsequently compared plaque formation to the corresponding wild type. We also included an NCTC12658*∆kpsM* mutant constructed in a previous study [15]. Here we found that CP81-type phages isolated on the NCTC12658 and NCTC12662 strains neither formed lysis nor plaques on the acapsular mutants, thus showing high dependency on the capsular polysaccharides for successful infection (Table 4). On the contrary, the CP220-type phages isolated on strain RM1221 all infected the acapsular NCTC12662*∆kpsM* mutant to near wild type levels, often also exhibiting clearer lysis and plaques (Fig. 4). Thus, the isolated CP220-type phages infect *C. jejuni* independently of the capsule presence.

**Table 4:** Phage host range of mutant *C. jejuni* strains.

**Phage**	**Indicator strain used for isolation**	**Number of plaques (log_10_) formed on strain**					
		**NCTC12658**	**NCTC12658∆*kpsM***	**NCTC12658*∆motA***	**NCTC12662**	**NCTC12662∆*kpsM***	**NCTC12662*∆motA***
F347	NCTC12658	7.7	R	7.6	7.7	R	7.7
F348	NCTC12658	9.2	R	9.4	9.0	R	9.4
F349	NCTC12658	8.1	R	8.2	8.5	R	8.1
F350	NCTC12658	7.3	R	6.5	7.2	R	7.2
F351	NCTC12658	7.1	R	7.0	8.3	R	8.0
F352	NCTC12658	7.8	R	7.8	8.1	R	8.3
F353	NCTC12662	7.6	R	7.2	9.3	R	9.3
F354	NCTC12662	6.8	R	6.9	7.8	R	8.6
F355	NCTC12662	7.5	R	7.0	8.2	R	8.9
F356	NCTC12662	6.4	R	6.3	7.8	R	8.9
F357	NCTC12662	5.6	R	4.4	7.3	R	7.4
F358	NCTC12662	8.1	R	7.5	8.6	R	8.5
F359	NCTC12662	6.4	R	6.7	8.2	R	7.2
F360	NCTC12662	8.2	R	7.7	8.5	R	8.8
F361	NCTC12662	8.0	R	7.5	8.6	R	8.1
F362	NCTC12662	7.7	R	7.3	8.5	R	7.9
F363	NCTC12662	6.5	R	6.6	7.7	R	7.7
F364	NCTC12662	8.3	R	8.7	8.7	R	9.7
F365	NCTC12662	7.8	R	7.6	8.6	R	9.0
F366	NCTC12662	8.0	R	9.1	9.2	R	9.4
F367	NCTC12662	8.8	R	9.3	9.8	R	9.9
F368	NCTC12662	7.5	R	8.9	8.9	R	9.6
F369	NCTC12662	8.3^a^	R	8.3	7.2	R	9.2
F370	NCTC12662	4.1^b^	R	7.5^c^	8.6	R	9.2
F371	NCTC12662	R	R	7.5^c^	8.7	R	9.0
F372	NCTC12662	7.7^b^	R	8.2^c^	8.6	R	9.0
F373	NCTC12662	5.0^b^	R	8,4^c^	8.7	R	9.3
F374	NCTC12662	7.4	R	7,8	8.2	R	8.3
F375	NCTC12662	8.2	R	9.4	9.7	R	9.7
F376	RM1221	R	R	R	8.7	8.1	R
F377	RM1221	R	R	R	8.7	8.4	R
F378	RM1221	R	R	R	8.6	7.6	R
F379	RM1221	R	R	R	9.5	8.0	R
F380	RM1221	R	R	R	8.8	8.1	R
F381	RM1221	R	R	R	8.3	7.8	R
F382	RM1221	R	R	R	7.7	7.5	R
F383	RM1221	R	R	R	8.1	7.4	R
F384	RM1221	R	R	R	8.2	8.0	R
F385	RM1221	R	R	R	8.4	8.1	R
F386	RM1221	R	R	R	9.0	8.9	R
F387	RM1221	R	R	R	8.8	7.8	R
F388	RM1221	R	R	R	9.6	8.7	R
F389	RM1221	R	R	R	8.7	8.6	R

R: resistant (determined as no plaque formation).

^a^Very small plaques were only observed in 1 out of 3 experiments.

^b^Very small plaques only observed in 1 out of 4 experiments.

^c^Very small plaques observed in 1 out of 2 experiments.

### The role of motility for phage infection

We recently showed that the CP81-type phage F341, which is able to infect *∆kpsM* capsule deficient mutants of *C. jejuni*, is a flagellotropic phage that relies on motility for successful infection [15]. To investigate whether motility is necessary for infection of the capsule independent CP220-type phages isolated in this study, we constructed a non-motile NCTC12662*∆motA* deletion mutant. This NCTC12662*∆motA* strain was used in a host range assay with all isolated phages together with an NCTC12658*∆motA* strain made previously [15]. Interestingly, the host range assay showed that both non-motile mutants (NCTC12662 and NCTC12658 *motA*) were completely resistant to all the CP220-type phages, as no lysis or plaque formation was observed (Table 4). Consequently, our CP220-type phages depend on motility for successful infection and can be classified as flagellotropic phages. In contrast, all the CP81-type capsular dependent phages infected the non-motile strains with EOPs identical to the wildtype strains (Table 4). Thus, *C. jejuni* RM1221 promoted isolation of CP220-type flagellotropic phages.

### Motility during phage isolation

**Figure 5:**
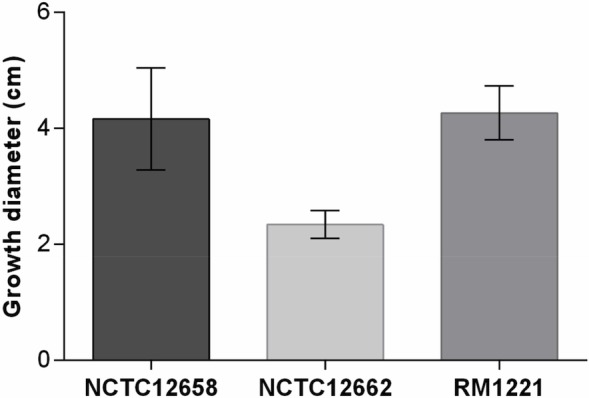
Motility of *C. jejuni* strains NCTC12658, NCTC12662 and RM1221 after 24 hours in low percentage agar. Motility was assessed as diameters, in centimetres, of growth zones after 24 and 48 h of incubation, only 24 h data is shown.

Noteworthy, all phages isolated in this study were able to infect the NCTC12662 strain with high EOPs, but only phages dependent on the capsule were isolated on this strain. Differences in rotary speed of flagella have previously been shown to have an effect on infection by flagellotropic phages [46,47]. Thus, we therefore speculated if differences in the motility of the three phage isolation strains might explain why no flagellotropic phages were isolated on NCTC12662. To determine this we tested motility of the NCTC12658, NCTC12662 and RM1221 strains by microscopy and by investigating their ability to swarm in soft agar. These results showed a significantly reduced swarming behaviour of NCTC12662 as compared to NCTC12658 and RM1221 (Fig. 5). Growth rates of all three strains in BHI broth were identical, demonstrating that NCTC12662 was indeed impaired in motility compared to NCTC12658 and RM1221. Also, we did observe that lowering the agar concentration from 0.6% to 0.5% or 0.4% in the top agar during plaque assays using NCTC12662 greatly enhanced plaque formation and visibility of CP220-type phages. Plaques formed on 0.6% top agar were small (approx. 0.5 mm in diam.) whereas 0.5% and 0.4% top agar resulted in medium and large size plaques (approx. 1–3 mm in diam.). Thus, the reduced motility of NCTC12662 may explain why no flagellotropic phages were isolated on this strain in spite of its sensitivity towards this group of phages.

## Discussion

Phages infecting *C. jejuni* have primarily been isolated from various chicken and poultry sites, due to the high colonisation level of the bacterium within the chicken gut [3,4,30–33,35–37,45,48–53]. A large group of these phages are dependent on the CPS structures expressed by *C. jejuni* in order to establish a successful infection [11,13,14]. Thus, with the aim of isolating novel phage types and investigating the role of the phage isolation strain during this process we used *C. jejuni* strains expressing different CPS structures for phage isolation from free-range chicken farms.

A total of 43 phages from seven different farms were isolated using three different *C. jejuni* strains. Characterisation of these phages showed that the phage diversity was restricted to two major groups that fall within the recently described phage types for *C. jejuni* based on genomic features and morphology, the “Cp220likevirus” and the “Cp8unalikevirus” [40]. These genera are represented by the two type-phages, *C. jejuni* phage CP220 having a genome of 178 kb and phage CP81 with a genome size of 133 kb, but both being myoviruses [40]. Surprisingly, in this study we found that the phage type correlated with the isolation strain. The two isolation strains NCTC12658 and NCTC12662 promoted the isolation of CP81-type phages, whereas only CP220-type phages where isolated on RM1221. These two phage types have been recovered exclusively over more than 20 years from poultry/chicken settings throughout the world [3,30–33,35–39,41,54–56] indicating that they, and their bacterial hosts, are highly adapted to this environment. Isolating true novel *C. jejuni* phage types may thus require exploring other niches than poultry settings where this bacterium can be found, although this has proven difficult [49,57]. Low concentration of phages and *C. jejuni* in other environments have been suggested as limiting factors for phage isolation, but perhaps the choice of isolation strains used in these experiments may also have been a contributing factor. Often the isolation strain has been of a different origin than the environment sampled such as *C. jejuni* NCTC12662 originating from chicken, due to its high phage sensitivity [57]. We have in this study shown that the applied isolation strain is of great importance for the phage type recovered from a given sample. Hence, using representative *C. jejuni* strains from the environment of interest or improving the knowledge of the surface composition expressed by such environmental isolates may aid in choosing more appropriate phage isolation strains and thus improve phage recovery from such locations.

Interestingly, we found that the *C. jejuni* phages isolated in this study were either dependent on CPS or motility for successful infection. Indeed, carbohydrate surface structures such as the capsule and lipopolysaccharides together with flagella are often recognised as receptors of bacteriophages [58]. A recent screening for phages infecting *Salmonella enterica* serovar Typhimurium also resulted in phages targeting only a limited number of receptor types, including flagella, LPS (lipopolysaccharide) and a membrane transport protein (BtuB) [59]. Noteworthy, we found that the dependency of CPS for successful phage infection correlated with both the isolation strain as well as the phage type, as our CP81-type phages were dependent on CPS for infection, while CP220-type phages relied on motility for successful infection. Using transposon mutants Coward and co-workers also observed that phage-resistance towards group III phages (CP81-type phages) were associated with mutations in the CPS locus, whereas resistant variants to group II phages (CP220-type phages) were non-motile [11]. While this correlation holds true for many *C. jejuni* phages, we have recently characterised a CP81-type phage F341 isolated on strain NCTC12685 that infects *C. jejuni* independently of the capsule, but instead requires a motile flagellum for infection [15]. Thus, this exception to the rule shows that the *C. jejuni* phage type (genome size) is not always linked to receptor type (CPS or flagella) and that the studies of phage receptors provide insight into *C. jejuni* phage diversity. In addition, more *C. jejuni* phage genome sequences are required to expand our understanding of the *C. jejuni* phage types, which currently is based on a limited number of phage genome sequences.

It is both striking and surprising that the *C. jejuni* NCTC12662 strain only promoted the isolation of one phage type that consistently relied on the capsule for infection, even though both the CPS- and motility-dependent phages isolated in this study proliferate to high numbers on this strain. NCTC12662 (also called PT14) is referred to as the universal *Campylobacter* bacteriophage host strain and has been extensively used for the isolation of phages infecting *C. jejuni* due to its general phage sensitivity [4,10,30–38,44]. Nevertheless, several studies also observed that predominantly CP81-type phages were isolated using NCTC12662 as indicator strain [32,33,35–37] supporting our observation that NCTC12662 primarily select for this phage type during the isolation process. However, one might also speculate that the CP81-phage types simply are more predominant in the chicken gut, hence more easily isolated, and we did indeed isolate twice as many phages of this type as compared to CP220-type phages. But interestingly, we discovered that NCTC12662 was impaired in motility as compared to NCTC12658 and RM1221 and we propose that the speed of the flagella rotation is a crucial factor during phage isolation, in particular when phages are embedded in faecal material and are present in relatively low numbers. Indeed, phages using the flagellum as the primary receptor are less frequently isolated from complex material compared to phages binding to the bacterial envelope [58]. Furthermore, it has been reported that the speed of flagella rotation influences the infection efficiency of the flagellotropic phage 7-7-1 infecting *Agrobacterium* [47]. A similar mechanism may apply for *C. jejuni* phages, as we recently showed that phage F341 require motile flagella to absorb and infect *C. jejuni* suggesting that the flagella rotation transports the phage to the bacterial body where the phage DNA is injected [15]. We also found that lowering the top agar concentration greatly enhances plaque sizes of CP220-type phages formed on NCTC12662. Thus, whether lowering the top agar concentration during the phage isolation process or using NCTC12662 mutants with enhanced flagella rotary speed will enhance the recovery of flagellotropic phages using this strain remains to be explored.

The *O*-methyl phosphoramidate (MeO*P*N) surface moiety is a common modification of the otherwise very diverse CPS structures expressed by many different *C. jejuni* strains and is implicated in important biological functions such as serum resistance and colonisation of piglets [27,28]. Interestingly, in the present study the *O*-methyl phosphoramidate (MeO*P*N) modification was the only common CPS structure of the strains sensitive to the CP81-type capsular dependent phages. Furthermore, we have previously shown that MeO*P*N of the CPS of *C. jejuni* NCTC11168 is a phage receptor recognised by many capsular phages [13,14]. Thus, it is likely that the capsular phages isolated in this study also rely on this structure for successful infection and we speculate that MeO*P*N comprises a general receptor of this group of *C. jejuni* phages. Interestingly, even though the *C. jejuni* 81–176 strain expresses MeO*P*N it was highly resistant to all phages isolated. This may be explained by its different CPS carbohydrate backbone structure as well as other phase variable modifications that may interfere with phage binding [21,60]. However, as 81–176 is resistant to all phages (i.e. both capsular and flagellotropic), the phage resistant phenotype may not be associated with receptor availability. Indeed, it has been demonstrated that *C. jejuni* 81–176 encodes a number of different restriction/modification systems that are absent in strains NCTC11168 and RM1221, which may degrade the phage DNA upon entry thereby inhibiting phage proliferation [61]. In *C. jejuni* NCTC11168, phage resistance is easily achieved by phase variation both *in vitro* and *in vivo* by changes in the polyG tract length located in the MeO*P*N transferase gene *cj1421* without affecting growth and colonisation ability of the bacterium [13,14]. Hence, becoming phage resistant by losing the receptor does not have an impact on the ability of *C. jejuni* NCTC11168 to grow and colonise. Also, the MeO*P*N capsular modification is phase variable in many other *C. jejuni* strains [18,19,21,22,24,25,27,60]. However, in NCTC12662 only one of the two MeO*P*N-transferase homologues shows phase variation at the sequence level [44]. Consequently, the ability not to switch off MeO*P*N or a lower switching rate could explain why *C. jejuni* NCTC12662 is consistently sensitive to many capsular phages, thus promoting isolation of this group of phages.

In conclusion, 43 novel phages infecting *C. jejuni* were isolated and 20 were further characterised to belong to the *Eucampyviriniae* subfamily either Cp220likevirus or Cp8unalikevirus. Interestingly, we found that the phage type correlated with the isolation strain as well as the receptor dependency, and only *C. jejuni* phages that were dependent on previously described receptors, namely the CPS and the flagella, were isolated. Thus, even though our attempts to isolate novel *C. jejuni* phage types in terms of receptor (and morphology) by using strains expressing different CPS structures were unsuccessful, our study emphasises the importance of isolation strain, since the repeatedly used *C. jejuni* NCTC12662 strain selected for one phage type with a strong bias towards one receptor type. It is well known that the chicken gut is the major ecological niche of *C. jejuni* providing the perfect environment for growth and colonization of this bacterium. Our study indicates that also *C. jejuni* myoviruses with limited receptor types and genome sizes are highly predominant and may be well-adapted to this ecological niche of *C. jejuni*. Identifying true novel *Campylobacter* phages may thus require exploring other *Campylobacter* hosts, such as *C. fetus* and *C. upsaliensis* and their niche environments in sheep, dog or cat, respectively.
